# Lay people expect social modernization will bring more societal well-being: the relation between expected societal development, communion, agency and subjective well-being

**DOI:** 10.1186/s40359-024-02142-5

**Published:** 2024-12-05

**Authors:** Mateusz Olechowski, Kuba Krys

**Affiliations:** Institute of Psychology, Polish Academy of Sciences, Warsaw, Poland

**Keywords:** Future, Modernization, Societal development, Wellbeing, Communion, Agency

## Abstract

**Background:**

Previous studies showed that lay people see modernization as a threat to social fabric because it will make people less warm and moral. The purpose of this paper is to describe lay people’s understanding of the effects of different types of modernization. Specifically, we checked how social, economic, technological and conventional development are expected to influence communion, agency and well-being in the future society.

**Methods:**

We conducted three cross-sectional studies using online surveys. Prolific participant pool users over 18 years of age that held Canadian citizenship and resided in Canada were eligible to take part in the study in exchange for financial compensation. T-tests and linear regression analyses were conducted using SPSS statistical package.

**Results:**

Participants expected that people in future society will have lower well-being than today. Technological modernization was expected to decrease communion and well-being but increase agency in the future, while social modernization was expected to strengthen societal communion, agency and well-being.

**Conclusion:**

Lay people believe that different types of modernization will have different effects on society. Whereas technological progress is viewed ambivalently, social development is seen as uniformly positive for well-being of society. In order to counter pessimism about the future, policy makers should focus on social development while striving to mitigate negative social aspects of technological advancements.

**Supplementary Information:**

The online version contains supplementary material available at 10.1186/s40359-024-02142-5.

## Introduction

According to recent research, people believe that over time their societies become more competent, but also less communal i.e. warm and moral [1–3]. Since communion is a crucial ’social fabric’ that holds people together, decreasing it might lead to societal plight. Such vision of the future is a recurring theme in lay discussions and can be illustrated by science-fiction famous dystopic blockbuster movie *Blade Runner*, where extraordinary technological advancements coexist with a deeply cynical, broken society. Such vision of humankind’s future is a bleak one but is it indeed the full picture of how people think about the future? Previous research on lay predictions focused mostly on a specific aspect of societal changes brought about by modernization – related mostly to technological progress, globalization and industrialization. It is therefore possible that other paths of societal development – like social development – might make people expect a different, more positive effect on society’s communion, and, by extension, on its well-being.

The purpose of this paper is to describe lay people’s predictions about how their societies will develop and how they will change in terms of traits and well-being depending on the development pathway these societies will undertake. We do it by describing people’s expectations about (a) developmental pathways their societies will choose, (b) communion and agency levels in future society, (c) well-being levels in future society, and (d) analyzing how these factors relate to each other. In doing so, this paper extends the current discussion on folk theory of social change and modernization by showing that different types of modernization differently relate to people’s expectations about future society, and – importantly - that some types of modernization are perceived as bringing only improvements. Such inquiry is worthwhile because in democratic systems people are important actors in societal progress. Although they cannot influence such macrotrends directly, they play a considerable role as citizens, voters and consumers. Understanding what they predict and fear is also crucial for policymakers and advocacy groups to better tailor their policy and communication to ease these fears and garner more social support for constructive citizen behaviour and positive societal changes.

### Future perception

Thinking about the future and taking it into account in present behaviour is one of the core human characteristics. According to evolutionary perspective, mental time travel (or prospection) - the capacity to mentally project oneself into the future - enabled early humans to prepare for potential threats, secure food resources, and create social alliances. This skill allowed humans to not only react to immediate environments but also plan for the longer term [4]. Because of this evolved ability, contemporary humans are able to set goals and devise strategies to achieve them, which is crucial for personal and professional success. According to goal-setting theory, setting specific and challenging goals for the future enhances motivation and performance in the present [5]. There are considerable individual differences in human’s attitudes and perceptions of the future. For example, more future-oriented individuals - those who plan and set long-term goals – achieve more positive outcomes, such as better academic performance, healthier behaviors, and better financial planning [6]. External factors can also influence future perception. Socioeconomic factors – for example – have been studied in this domain. People of lower social class have a more holistic way of thinking and therefore predict the future in a less linear way than people from the middle class [7]. Working class people – having fewer financial resources – feel that they have less control over their lives which can also influence their orientation towards the future, e.g. they are less prone to delay gratification [8, 9].

### Macro changes and modernization

Classic theories explaining societal development focused mainly on technological and economic progress. According to prominent Modernization Theory [10], societal evolution occurs through distinct phases of economic growth – from preindustrial economies through industrialization to creation of mass consumption society that focuses on living standards. Originating in the mid-20th century, this perspective mirrors an era wherein Western nations, notably the United States, were regarded as exemplars of economic and societal advancement. This viewpoint advocates that by emulating the developmental trajectory of Western industrial nations, other societies can attain similar levels of growth and wealth. This and similar views were later challenged. Post-Development Theory [11, 12] – among others – critically reevaluated established notions of development. Emerging in the late 20th and early 21st centuries, this theoretical approach contests the prevalent development models, highlighting their tendency to impose Western standards and values on varied cultural contexts, which can lead to outcomes such as ecological harm and social inequality. Other notable examples include Human Development, Sustainable Development and Social Development theories. What they all had in common is that they disagreed with the centrality of economic development as a measure of society’s progress. More recently psychologists joined this trend by bridging cross-cultural psychology with developmental studies and introducing the idea “cultural sensitivity” to indexes of societal progress [13, 14]. According to this conceptualization, societies’ progress should be measured based on their own culturally specific idea of what constitutes a “good life” for its members. To empirically support this view, Akalisky et al. [15] showed that values – in this case “freedom” – can be differently understood in the protestant West and in East Asia and that this could impact their developmental pathways. Similarly, Yin et al.’s [16] analyses suggest that the relation between subjective well-being and the Human Development Index (HDI) varies globally - the three HDI components (gross national income, education, life expectancy) only matter equally in Western and rich countries.

Regardless of how we view it, modernization brought unprecedented changes for human societies worldwide [17–19]. According to recent estimates, during the last century people’s life expectancy increased around 40 years, world’s gross domestic product grew 25 times and the computational capacity of personal computers doubles every 2 years. However, this progress did not come without its troubles. There’s a spreading awareness that today’s rapid economic growth and consumerism culture - that started with industrialization - is a growing menace to social fabric and local natural environments as well as global ecosystem. Similarly, exponential technological advancement like automatization and digitization – even though generally viewed as positive – makes some people anxious about changes it might bring to their lives, e.g. in the work domain.

Psychological studies support the idea that the world has been changing considerably in the recent decades. Many societies shift towards slower life history strategy which involves fewer offspring and more investement in children as well as focus on more long-term goals [20]. Individualism has also been shown to have risen in most societies around the world [21]. Well-being has been shown to be on the decline in the recent decades, at least in the United States [22]. Some studies have linked this negative trend to increasing use of new media like social media and smartphones [23]. Some experts theorize that these macro changes might be exacerbated in the future because of possible changes that technology might bring to the neural structure of people’s brains. Specifially, it has been suggested that hippocampus might decrease in volume as navigational technology becomes widely accessible and commonly used [24]. It has also been predicted that amygdala reactivity might increase which can have negative consequences for mental health like higher anxiety and depression [25]. Expert predictions about the future are further complicated by the COVID-10 pandemic. Many experts agree that the consequences can be profound. One of the most commonly expected changes is a negative change in well-being [25]. On the other hand though, the negative changes in well-being might be different according to geography as happiness has been shown to be on the rise around the world [26]. This suggests that at least this negative trend might be more important in the more economically and technologically advanced societies. Such a mixed and complex bag of changes and possible consequences raises the question of what are lay people’s predictions about the influence of societal changes on future societies.

### Lay theorizing on modernization

Theorizing and research on *prescribed or actual* processes of societal changes, on a variety of dimensions – economic, technological, social, cultural, psychological - is abundant. Recently, psychology started contributing to this important field by studying *folk theorizing* regarding modernization, including people’s perceptions and expectations [1, 27]. According to *Folk Theory of Social Change* (FTSC) the process of modernization is seen as a natural progression from ‘traditional society’ to ‘modern society’ [1]. According to this, in ‘traditional society’ resources are shared between community members according to their needs, and that is contrasted with ‘modern society’ with impersonal exchange relationships, where goods and services are traded in a monetary system. Traditional society was theorized to be seen as more communal (but less agentic) and modern society as more agentic (but less communal). In this emerging field of research, it was repeatedly documented that lay people perceive modernization as bringing both positive and negative effects on social fabric – in the long run modernization is perceived as increasing societal competence, but decreasing communion [1–3]. This phenomenon was also found to be similar across countries and cultures. In a study conducted in United States, Australia, New Zealand, Austria, the Philippines, China, and Malaysia, people in all countries expected their society to become more agentic over time. In the majority of these nations, individuals also anticipated a decrease in communion of their societies, although this trend was minor and not statistically significant among people from New Zealand and the United States [28]. However, the aforementioned studies relied mostly on technology- and industrialization-related understandings of modernization. Therefore, it cannot be determined with confidence that these effects would hold for other types of modernization.

### Communion, agency and well-being

If such a folk theory has taken a hold on the public imagination around the world, then this is highly consequential as these evaluations can have a considerable influence on what we think, how we feel and how we behave toward others [29, 30]. Agency and communion are two fundamental dimensions of social cognition – people commonly and automatically evaluate others on how communal and how agentic they are [31, 32]. Agency refers to goal-achievement and task functioning, whereas communion refers to the maintenance of relationships and social functioning [29]. Both dimensions were further differientiated into two facets each – agency into competence and assertiveness and communion into warmth and morality [33].

One crucial difference between agency and communion is whether they primarily benefit oneself (agency) or other people (communion). Therefore, people see agency as more desirable and significant when looking at themselves, while communion is viewed as more desirable and significant when considering others because it’s in people’s self-interest [32, 34, 35]. In line with these results, expected communion (but not agency) in future society, was consistently associated with support and willingness to enact this future change [3]. In other words, people wanted to live in a future society that is high on communal (but not necessarily agentic) traits. However, these studies did not differentiate between facets of agency (competence and assertiveness) and they presented vignettes with a hypothetical future (e.g. marihuana is legalized) instead of asking about the actual future. They also did not assess the expected consequences of communion (or agency) for the future society like it’s predicted well-being.

Well-being is a complex, multidimensional concept that includes various aspects of individuals’ lives, including their emotional, psychological, social, and physical health. This multifaceted nature of well-being can be understood through different theoretical frameworks. In the hedonic well-being perspective, rooted in the philosophy of hedonism, the focus is on pleasure attainment and pain avoidance. It is often measured through life satisfaction and affect balance [36]. Eudaimonic well-being – on the other hand - stemming from Aristotelian philosophy, emphasizes living in accordance with one’s true self and realizing one’s potential. Ryff’s [37] model of Psychological Well-Being is a prominent example of this perspective. Recently, there has been attempt to integrate these different theoretical approaches and measure various types of well-being in order to capture it in its complexity [38]. It has been argued that instead of focusing on happiness - or any other specific type of well-being - we should treat different types of well-being – satisfaction with life, meaning in life, harmony, spirituality – as interconnected.

## Present research

In a series of three correlational studies we assessed lay people’s predictions about the changes in development, traits and well-being of the future society. Specifically, we aimed at checking whether different types of development - conventional, social, economic and technological – are differently perceived in terms of societal communion, agency and well-being. Our research contributes to the literature in three main ways. While previous studies focused mostly on the technological type of modernization, we broadened the scope by including other relevant types of development. Other novelty is that while previous research focused mainly on communion and agency, we decided to measure all four subtypes of communion and agency – warmth, morality, competence and assertiveness. Lastly, our studies are first in measuring possible consequences of expected communion and agency in society, namely societal well-being. These changes enabled us to paint a more complex and – crucially – more realistic picture of lay perceptions of modernization. We chose to focus on Canadian samples because – as in the United States – Canada is an economically and technologically advanced society that has seen considerable decrease in well-being in recent times, especially among its youth [39]. We therefore wanted to check whether these negative trends can be seen in lay perceptions about society’s future.

In Study 1 we preliminarily tested the relation between expected communion, agency and well-being. In Study 2, we replicated previous results and added questions about expected societal development. Finally in Study 3, we confirmed previous results and extended them by testing for mediation effects. We hypothesized that expected communion and – to a lesser extent – agency, will positively correlate with expected well-being (Hypothesis 1). Secondly, we assumed that technological development will positively correlate with agency but negatively with communion (Hypothesis 2) and that social development will positively correlate with communion and agency (Hypothesis 3). Thirdly, we expected that communion and agency will mediate to some extent the relationship between technological and social development and well-being (Hypothesis 4).

## Method

### Procedure and participants

Data was collected in three correlational studies by means of an online questionnaire. Participants were recruited through Prolific, an online behavioral research platform. Recent studies suggest that Prolific provides consistently high-quality data [40, 41]. Prolific users over 18 years of age that held Canadian citizenship and resided in Canada were eligible to participate in the studies and were shown the description of the study in their Prolific panel. Those who decided to participate were redirected to the questionnaire on Qualtrics survey platform. Upon completion, subjects were paid for their participation through Prolific. To increase data quality, we employed several attention checks throughout the survey by asking participants to choose a specific answer. We excluded data from participants who failed one or more attention checks, which indicated inattentive responding [42, 43]. Table 1 shows the demographic characteristics of the final samples.

**Table 1:** Participants’ demographic characteristics

	*N*	Gender			Age		
		Female	Male	Other	Range	M	SD
Study 1	111	46%	52%	2%	18–63	33.41	11.08
Study 2	213	60%	39%	1%	18–68	33.85	11.60
Study 3	285	56%	41%	3%	18–76	35.34	12.37

### Measures

If not stated otherwise, all questions were asked about the state of Canadian society in the year 2060 compared to today (similar to Bain et al. [3]). They were answered on a scale from − 5 (much less than today) to + 5 (much more than today) with 0 (no different from today) as middle answer.

***Predicted communion and agency*** (Study 1, 2 and 3) [33]. Communion scale consisted of six items (Cronbach’s α = 0.88; 0.92; 0.91, for Studies 1, 2 and 3, respectively) – three about Warmth (e.g. empathetic) and three about Morality (e.g. fair). Agency scale also consisted of six items (α = 0.83; 0.88; 0.87) – three about Competence (e.g. clever) and three about Assertiveness (e.g. self-confident). Sample item read: *People in Canadian society in 2060 will be honest.*

***Predicted well-being*** (Study 1, 2 and 3). Well-being was measured with items derived from four scales, representing four aspects of well-being: (1) life satisfaction (Riverside Life Satisfaction Scale; 6 items) [44], (2) harmony in life (Harmony in Life Scale; 4 items) [45], (3) meaning in life (Meaning in Life Questionnaire – Presence Subscale; 4 items) [46] and (4) spiritual well-being (Spiritual Well-Being Questionnaire SHALOM, transcendental domain; 4 items) [47]. Sample item read: *People in Canadian society in 2060 will be satisfied with where they are in life.* For analyses we used a composite score of all 18 items (α = 0.90; 0.88; 0.87).

***Predicted societal development*** (Study 2 and 3; based on Krys et al. [48]). 33-item scale tapping into expectations about four broad types of modernization: (1) Conventional (five items; e.g. military power, traditions, high birth rates; α = 0.58; 0.62), (2) Economic (eight items; e.g. prosperity, productivity, stable prices; α = 0.81; 0.86), (3) Technological (five items; e.g. digitalization of everyday life, investing in science and technology, access to high-speed Internet; α = 0.79; 0.82) and (4) Social (15 items; α = 0.96; 0.96). The latter can be further divided into four subtypes: (a) Communal foundations (social trust, bonds between people, supporting family life), (b) Agentic foundations (health, democracy, safety, justice), (c) Welfare (eradicating poverty, education, work-life balance, freedom) and (d) Inclusivity (equality, human rights, openness to immigration). Sample item read: *I predict that in 2060*,* Canada will provide high quality education*.

Full item list for all measures can be found in SOM.

### Analysis

All statistical analyses were conducted using SPSS software (version 29.0). After initial inspection of descriptive statistics and sample characteristics, we conducted one sample t-tests for all measures to see whether their means differ significantly from the middle scale point. We also calculated corresponding effect sizes (Cohen’s *d*). Secondly, we ran a series of linear regression analyses with communion and agency predicting well-being and expected development predicting communion, agency and well-being. Finally, we used PROCESS, a specialized macro for SPSS based on linear regression [49], to test for indirect effects (mediation) between variables.

## Results

Across all three studies, communion was expected to stay the same and agency was expected to increase (Cohen’s *d*s > 0.16) in the future. However, this increase was driven fully by the agency-competence facet which was predicted to rise (all *d*s > 0.25), whereas assertiveness facet was not expected to increase. For communion, no difference emerged between Warmth and Morality facets. Also across all studies, participants predicted a decrease in well-being (*d*s < − 0.37). Across both Studies 2 and 3 - participants predicted that future will bring a significant development in the digitalization, science and technology domain (*d*s > 1.40). Also as expected, conventional development was believed to decrease (*d*s > 0.53) and economic development was believed to increase (*d*s > 0.17). Interestingly though, social development was predicted to stay on the same level as today. However, when we look at subtypes of social development, we can see that Communal foundations are expected to decrease (*d*s < 0.13) and Inclusivity is expected to increase (*d*s > 0.23) in the future.

To test Hypothesis 1, we regressed well-being on communion and agency. Results showed that expected communion was a strong, positive predictor of expected well-being across all studies (Study 1: *β* = 0.63, *p* < .001; Study 2: *β* = 0.50; *p* < .001; Study 3: *β* = 0.61, *p* < .001). On the other hand, agency emerged as a weaker and less reliable – albeit also positive – predictor of well-being (Study 1: *β* = 0.11, *p* = .15 ; Study 2: *β* = 0.20; *p* < .05; Study 3: *β* = 0.16, *p* < .01). These results suggest that the higher people predict communion and – to a lesser extent – agency levels in the future society, the more well-being in this society they expect. In order to assess whether these effects were driven by specific facets of communion and agency, we repeated the analyses with warmth, morality, competence and assertiveness as predictors. Results showed that communion facets - warmth and morality – as well as agency-assertiveness were reliably predicting expected well-being (see Table 2). No effect has been found for agency-competence.

**Table 2:** Regression coefficients for expected communion and agency predicting expected well-being

	Communion			Agency			
	Full scale	Warmth	Morality	Full scale	Competence	Assertiveness	
Study 1	0.63***	0.20*	0.39***	0.11	0.03		0.07
Study 2	0.50***	0.36***	0.14	0.20*	0.03		0.21*
Study 3	0.61***	0.32***	0.30***	0.16**	− 0.04		0.24***

* *p* < .05; ** *p* < .01; *** *p* < .001

In order to test Hypotheses 2 and 3, we ran three separate regression analyses with expected development as predictors and expected communion, agency and well-being as dependent variables, respectively (see Table 3). As expected, in both studies expected technological development was positively – albeit weakly – related to expected agency (*β* = 0.13, *p* < .05; *β* = 0.11; *p* < .05) but negatively to expected communion (*β* = .-15, *p* < .01; *β* = − 0.14; *p* < .001) in society. On the other hand, in both studies expected social development was strongly and positively related to expected communion and not related to expected agency. For well-being, the only reliable predictor across both studies was expected social development (*β* = 0.54, *p* < .05; *β* = 0.78; *p* < .05). We ran another regression analysis in which we substituted social development with its four subtypes. This revealed that the only two subtypes of social development were reliably predicting expected well-being were communal foundations (*β* = 0.40, *p* < .001; *β* = 0.26; *p* < .001) and welfare (*β* = 0.27, *p* < .05; *β* = 0.36; *p* < .001). For detailed results see SOM.

**Table 3:** Regression coefficients for expected development predicting communion, agency and well-being

	Communion		Agency		Well-being	
	Study 2	Study 3	Study 2	Study 3	Study 2	Study 3
Conventional	− 0.05	0.03	0.06	0.01	0.11	0.05
Economic	− 0.01	− 0.07	0.05	0.14*	0.06	0.04
Technological	− 0.15**	− 0.14***	0.13*	0.11*	− 0.05	− 0.11*
Social	0.36***	0.58***	− 0.08	− 0.10	0.54***	0.78***

Multiple regression analyses with communion, agency or well-being as dependent variable and types of societal development as predictors. Communion and agency were mutually controlled in the analyses to account for shared variance. Scores are standardized beta coefficients. * *p* < .05; ** *p* < .01; *** *p* < .001

In order to test Hypothesis 4, we ran regression analyses testing indirect effects using PROCESS macro for SPSS [49] with expected social and technological development as predictors, expected communion and agency as mediators and expected well-being as dependent variable. We found an indirect positive effect of social development on well-being through communion (IE = 0.09; SE = 0.03; CI = [0.04, 0.25]). We also found – although smaller – indirect effects of technological development on well-being through both communion (negative effect; IE = − 0.03; *SE* = 0.01; CI = [-0.06, − 0.01) and agency (positive effect; IE = 0.02; *SE* = 0.01; CI = [0.00, 0.04). Thus, we found evidence for Hypothesis 4 in the form of partial mediation effects.

## Discussion

In partial accordance with the FTSC literature [1–3], our study suggest that lay people expect an increase in agency in future society (but no changes in communion). What’s important, this expected change in agency was significant only for competence facet of agency but not assertiveness facet. This is a novel and noteworthy finding because in previous research on FTSC [1–3] agency was measured and analyzed as a unified concept, thus obscuring the more complex picture of results. On the other hand, we found that laymen expect a decrease in societal well-being. This suggests (WEIRD) people hold a rather dystopian vision of the future, akin to the one depicted in *Blade Runner*. We also found that people expect well-being of society to be related to its communion and agency levels – the more communal and agentic future society, the higher its well-being. What’s crucial though is that this relationship was significant only for the assertiveness facet of agency and not the competence facet. In other words, assertiveness and leadership skills are considered a valued resource supporting the well-being of a society, whereas intelligence and effectiveness are not. That agency-competence is the only facet of Big Two (communion/agency) that is not related well-being is important because it is also the only facet that is expected to go up in the future society. These results add to the literature on fundamental content dimensions [33].

Participants also predicted the future will bring a massive digital, scientific and technological development. In line with FTSC research [1], our results also showed lay people expect that in a more digitally, scientifically and technologically developed society, people will be more agentic but also less communal. On the other hand – extending the literature on FTSC because this was not studied in previous research – if people expect that society will develop in the social areas (especially by addressing poverty, foster trust and bonds between people, support work-life balance and education), they actually expect higher communion level. What’s additionally interesting, social development – unlike technological development – was not predicted to change (increase or decrease) in the future. This might suggest that social development is not consensually considered an inevitable part of modernization (as technological development) but rather a political issue based on people’s (and elites’) preference and choice. Results of these studies suggest that when studying modernization it’s imperative to take into account the diversity of different possible developmental pathways as well as complexities of fundamental content dimensions, which was not done in the previous studies [1–3].

Based on these results, several practical suggestions can be proposed. Considering people’s pessimistic outlook towards future societal well-being, initiatives should focus on highlighting the positive trends in societal development. This would help in fostering a more optimistic and realistic view of the future and countering feelings of helplessness and cynicism. Moreover, as advancements in technology are predicted to increase people’s agency but potentially reduce communal bonds, political efforts should be made to ensure that technological growth supports, rather than undermines, social connections. Finally, our findings point to the significance of advocacy - among both the public and policymakers - about the long-term benefits of investing in social infrastructure, focusing on how these investments contribute towards optimism about society’s future.

### Limitations and future directions

Our research comes with several important limitations. Firstly, because of the correlational nature of the studies, causal relation between variables – and its direction – cannot be conclusively established. We hypothesized that people’s communion and agency predictions influence their well-being predictions. We also assumed that it is the expected societal development that leads to different visions of communion, agency and well-being in society. However, the opposite can be true, e.g. participants might predict well-being in future society and then base their inferences about communion and agency in people on that well-being level. It is also possible that the relation between variables is reciprocal, e.g. communion and agency predictions might influence societal development predictions and societal development predictions might influence communion and agency predictions. Secondly, for all reported studies, only Canadian participants were sampled which significantly limits the generalizability of results and scope of conclusions. There is ample evidence that WEIRD (Western, Educated, Industrialized, Rich, Democratic) countries – like Canada – differ on many numerous psychological dimensions from the majority of world population [50]. This might be especially relevant when studying lay perception of societal development as this issue has been shown to be highly variant across cultures [48]. Exploring how different cultures impact the interplay between predicted communion, agency, and well-being might be a promising research avenue. Thirdly, studying participants’ predictions of future events comes with its own problems. Specifically, people’s forecasting has been shown to be incorrect in many cases, due to various biases (e.g [51]). The vision of the future can also be influenced by one’s beliefs about the present – in the case of this research these would be, for example, evaluations of today’s society communion, agency and well-being. What’s more, forecasting can be also affected by participants’ beliefs about their own levels of communion, agency and well-being. It would be beneficial to investigate – or at least control for – these variables in future research.

Knowledge on the topic in question is still modest and requires more research. For example, our findings suggest that people expect a rise in the competence aspect of agency but not assertiveness. Further investigations could focus on why assertiveness appears more crucial for societal well-being than competence. Another potential area that remains underexplored is the exact ways in which communion and agency affect predicted well-being of society. Gaining better understanding of these mechanisms could offer insights for crafting strategies to increase support for positive change. Finally, conducting longitudinal studies would allow observing shifts in communion, agency and well-being and could lead to a more nuanced understanding of their interplay. Such studies would be also valuable for pinpointing potential risk and protective factors that may affect people’s perceptions of the future. Additionally, with observations that individuals anticipate a decline in societal well-being linked to communion and assertiveness levels, future research could look into what contributes to such pessimistic future outlooks and possible ways to mitigate them. Lastly, our studies suggest that technological advancement is expected to increase agency but decrease communion. Future research could focus on finding out what specific type or aspect of technological changes drive these effects.

## Electronic supplementary material

Below is the link to the electronic supplementary material.

Supplementary Material 1

## Data Availability

Data available on request from authors.

## Abbreviations

SOM: Supplementary Online Material
