# Supplementary material for: Lay people expect social modernization will bring more societal well-being: the relation between expected societal development, communion, agency and subjective well-being
Source: BMC Psychol. 2024 Dec 5;12:729. doi: 10.1186/s40359-024-02142-5 (PMC11619630; doi:10.1186/s40359-024-02142-5)
Supplement: Supplementary file 1 — Supplementary Material 1 [file 40359_2024_2142_MOESM1_ESM.docx]

**Supplementary Online Materials**

**Method**

**Measures**

**Communion and agency scales**

| **Dimension** | **Facet** | **Items** |
| --- | --- | --- |
| Communion | Warmth | Warm, empathetic, unfriendly, uncaring |
| Communion | Morality | Honest, just, unfair, untrustworthy |
| Agency | Competence | Competent, resourceful, intelligent, uncapable, unclever, inefficient |
| Agency | Assertiveness | Self-confident, having leadership abilities, giving up very easily, going to pieces under pressure |

**Riverside Life Satisfaction Scale (6 items).** Please estimate how**satisfied** people in Canadian society will be **with their lives** in 2060 compared to today**. People in Canadian society in 2060 will...**

1. like how their lives are going.
2. be content with their lives.
3. be satisfied with where they are in life right now.
4. want to change many things in their lives (if they could live their lives over).
5. have the feeling that those around them seem to be living better lives than they are.
6. want to change the path their lives are on.

**Harmony in Life Scale (4 items).** Please predict to what extent people in Canadian society will live in harmony and balance in 2060 compared to today. People in Canadian society in 2060 will...

1. believe most aspects of their lives are in balance.
2. feel they are in harmony.
3. accept the various conditions of their lives.
4. fit well with their surroundings.

**Meaning in Life Questionnaire - presence subscale (4 items).** Please estimate how much meaning and purpose in life will people in Canadian society have compared to today. People in Canadian society in 2060 will**...**

1. not understand their lives’ meaning.
2. have a good sense of what makes their lives meaningful.
3. have discovered a satisfying life purpose.
4. live lives with no clear purpose.

**Spiritual Well-Being Questionnaire SHALOM - transcendental domain subscale (4 items).** People differ in their spirituality level and relation with the transcendent. The transcendent is some-One or some-thing independent of material universe and beyond known physical laws. It can be God, ultimate concern, cosmic force or other transcendental reality. Transcendence can be experienced through an established religion as well as outside of any religion. Please estimate how much relation with transcendent will people in Canadian society have in 2060 compared with today. People in Canadian society in 2060 will...

1. have little personal relationship with transcendent.
2. not be interested in the worship of transcendent.
3. feel oneness with transcendent.
4. feel at peace with transcendent.

**Predicted societal development scale (34 items).** We would like to ask some questions about how Canada might change in the future. Below you will find different possible aims of development. Please rate to what extent Canada will be reach these aims in 2060 in comparison to today. Don't answer based on how you want it to change. Answer based on how you predict it will actually change. In 2060 Canada will...

| **Type of development** | **Subtype of development** | **Items** |
| --- | --- | --- |
| Conventional | - | Emphasize religious values; have strong military power; have high birth rates; emphasize Canadian traditions; ensure people respect the authorities. |
| Economic | - | Maximize economic prosperity; develop industry; open new factories; manufacture goods; support the development of companies; foster international trade; keep stable prices; keep the Canadian dollar strong. |
| Technological | - | Provide common access to fast internet; enable people to complete official procedures on-line; enable people to work and study remotely; invest in new technologies; invest in science. |
| Social | Communal foundations | Make life easier for families; build trust between people; have strong social ties among people. |
| Social | Agentic foundations | Ensure a healthy and long life for its citizens; have a well-functioning democracy; make people feel safe in the streets; have a well-functioning justice system. |
| Social | Welfare | Get rid of poverty; provide high quality education; care for work-life balance; ensure its citizens live in freedom. |
| Social | Inclusivity | Be accepting of people coming to live in Canada from other countries; successfully fight all forms of inequality; protect human rights; successfully fight inequality between men and women. |

**Results**

Table 1. Effect sizes (Cohen’s *d*) for predicted communion and agency and their facets

|  | Communion | | | Agency | | | |
| --- | --- | --- | --- | --- | --- | --- | --- |
|  | Full scale | Warmth | Morality | Full scale | Competence | Assertiveness | |
| Study 1 | .01 | -.01 | .03 | .27** | .45*** | | .04 |
| Study 2 | -.09 | -.07 | -.10 | .16* | .25*** | | .03 |
| Study 3 | -.06 | -.05 | -.06 | .16** | .30*** | | -.02 |

* *p* < .05; ** *p* < .01; *** *p* < .001.

Table 2. Effect sizes (Cohen’s *d*) for predicted well-being and its facets

|  | Composite measure | Satisfaction | Harmony | Meaning | Spiritual |
| --- | --- | --- | --- | --- | --- |
| Study 1 | -.43*** | -.63*** | -.17 | -.22* | -.35*** |
| Study 2 | -.40*** | -.52*** | -.14 | -.13 | -.50*** |
| Study 3 | -.37*** | -.51*** | -.19** | -.15* | -.33*** |

* *p* < .05; ** *p* < .01; *** *p* < .001.

Table 3. Effect sizes (Cohen’s *d*) for development types

|  | Conventional | Economic | Technological | Social |
| --- | --- | --- | --- | --- |
| Study 2 | -.63*** | .17* | 1.40*** | -.07 |
| Study 3 | -.53*** | .26*** | 1.69*** | .06 |
|  | Communal foundations | Agentic foundations | Welfare | Inclusivity |
| Study 2 | -.28*** | -.17* | -.04 | .23*** |
| Study 3 | -.13* | .05 | .07 | .27*** |

* *p* < .05; *** *p* < .001.

Table 4. Regression coefficients for development types predicting well-being

| predictors DV | Well-being | |
| --- | --- | --- |
|  | Study 2 | Study 3 |
| Conventional | .10 | .04 |
| Economic | .05 | .05 |
| Technological | .00 | -.11* |
| Social (Communal foundations) | .40*** | .26*** |
| Social (Agentic foundations) | -.17 | .06 |
| Social (Welfare) | .27* | .36*** |
| Social (Inclusivity) | .06 | .14 |

Table 5. Regression coefficients for communion and agency predicting well-being and its facets

|  |  | Composite measure | Satisfaction | Harmony | Meaning | Spirituality |  |
| --- | --- | --- | --- | --- | --- | --- | --- |
| Study 1 | Communion | .63*** | .55*** | .59*** | .38*** | .36** |  |
|  | Agency | .11 | .07 | .09 | .34*** | -.19 |  |
| Study 2 | Communion | .50*** | .54*** | .30** | .52*** | .09 |  |
|  | Agency | .20* | .03 | .28** | .15 | .12 |  |
| Study 3 | Communion | .61*** | .51*** | .61** | .51*** | .16 |  |
|  | Agency | .16** | .13 | .10 | .20** | .08 |  |

Table 6. Regression coefficients for facets of communion and agency predicting well-being and its facets

|  |  | Composite measure | Satisfaction | Harmony | Meaning | Spirituality |
| --- | --- | --- | --- | --- | --- | --- |
| Study 1 | Warmth | .20* | .25* | .24* | -.01 | .29* |
|  | Morality | .39*** | .34* | .41** | .44*** | .07 |
|  | Competence | .03 | .04 | .11 | .11 | -.19 |
|  | Assertiveness | .07 | .03 | -.03 | .23* | .00 |
| Study 2 | Warmth | .36*** | 40*** | .22* | .36*** | .07 |
|  | Morality | .14 | .14 | .08 | .16 | .01 |
|  | Competence | .03 | -.13 | .01 | .07 | .14 |
|  | Assertiveness | .21* | .18 | .30** | .12 | .01 |
| Study 3 | Warmth | .32*** | .23** | .31*** | .34*** | .06 |
|  | Morality | .30*** | .29*** | .32*** | .18* | .12 |
|  | Competence | -.04 | .01 | -.05 | .00 | -.09 |
|  | Assertiveness | .24*** | .14 | .15* | .24*** | .18* |

Figure 1. Path model with the relationship between development and well-being partially mediated by communion and agency (Study 2)

Social development

Communion

Well-being

Technological development

Agency

.13**

.34***

-.15***

.18*

.34***

-.04

-.01

.61***

.29***

Standardized regression coefficients; **p < .01, ***p < .001. The direct effect is reported above the line, the total effect below the line.
